# Dynamic performance of a slab track–concrete box subgrade under a double-line high speed railway

**DOI:** 10.1371/journal.pone.0311969

**Published:** 2024-12-17

**Authors:** Jinglei Liu, Weihao Zhou, Jinyuan Cao, Xiuxin Li, Yinghui Jin, Qingzhi Ye, Guishuai Feng

**Affiliations:** 1 Hebei University of Architecture, Zhangjiakou, Hebei, China; 2 Hebei Key Laboratory of Diagnosis, Reconstruction and Anti-disaster of Civil Engineering, Zhangjiakou, Hebei, China; 3 Hebei Innovation Center of Transportation Infrastructure in Cold Region, Zhangjiakou, Hebei, China; 4 School of Civil Engineering, Southwest Jiaotong University, Chengdu, China; University of Sharjah, UNITED ARAB EMIRATES

## Abstract

Concrete box subgrades constructed from reinforced concrete serve as alternatives to conventional fill subgrades, effectively addressing the scarcity of high-quality fill materials. A hybrid simulation approach that merges coupled dynamics with finite element modelling was adopted for both single-line and double-line ballastless track-box subgrade systems, enabling a comparative analysis of dynamic stress, displacement, and acceleration. The results reveal that, when the two traffic conditions are compared, the dynamic response of the concrete box subgrade under double-line opposing operation shows a marked increase, particularly when the dynamic displacement increases by 80%. Under opposing traffic conditions, the dynamic stress on the subgrade surface exhibits a "saddle" distribution. Vertically, the dynamic stress inversely increases within the roof and rapidly attenuates in the vertical web and floor, with reductions reaching 92.7% at the floor bottom, demonstrating the substantial capacity of the concrete box subgrade to disperse train loads. The peak dynamic displacements recorded at the subgrade surface are 0.178 mm for single-line traffic and 0.320 mm for opposing operations, indicating minimal overall vertical deformation of the concrete box subgrade. Notably, the dynamic displacement on the subgrade surface results primarily from the underlying weak subsoil. Vertical acceleration attenuation occurs predominantly within the vertical web depth, with attenuation rates exceeding 95%. The environmental vibrations induced by high-speed trains predominantly affect the area within 0 to 4 m from the edge of the subgrade floor.

## 1 Introduction

High-speed railways have gained considerable attention worldwide, providing a convenient mode of travel by connecting cities and reducing travel time. The embankment structure, a crucial element of the substructure in high-speed railway systems, is essential for maintaining the safety and comfort of train operations [1]. This necessitates the application of stringent design standards and requirements for subgrade structures. To investigate the dynamic performance of conventional subgrades, numerical simulations, alongside field testing and model tests, serve as highly precise approaches for the study of subgrade dynamics, concurrently facilitating research into innovative subgrade design.

Initially, technological constraints limited research to 2D models for investigating train vibration issues [2]. Therefore, more sophisticated models have emerged, such as 2.5D [3–10] and the thin-layer method (TLM) [11, 12], increasing computational efficiency. Bian XC et al. [13–15] employed a 2.5D finite element model combined with Fourier transform techniques to explore resonance in track structures and subgrades under the impact of train loads, a critical aspect for operational safety and stability. Their research underscored the potential resonance effects induced by high-speed trains, which pose risks to operational safety and subgrade stability. Costa PA et al. [16, 17] used a 2.5D FE-BE coupled model for vibration analysis due to train loads and aligned their findings with real-world data, thus validating the model’s feasibility. Their research also highlighted the significant impact of factors such as stiffness and damping on wave propagation. Despite the broad application of 2.5D models, their limitations necessitate the development of 3D models [18–20]. Galvín P et al. [21, 22] introduced a comprehensive 3D analysis model to investigate vibration issues in both ballasted and ballastless track subgrade structures, focusing on the dynamic characteristics of transition zones. El Kacimi A et al. [23] leveraged a 3D finite element model, integrating the train and track while accounting for material damping, to assess dynamic responses at varying speeds. Varandas JN et al. [24] implemented a 3D finite element model to explore the causes of settlement in transition zones of tracks, ballast, and embankments. Correia AG et al. [25] incorporated the nonlinear properties of soil materials in their model, enhancing the prediction accuracy of subgrade dynamic responses. Wang J et al. [26] studied the dynamic response of a superthick backfill roadbed under high-speed railway loads.

The prevalent design for railway subgrade structures is a layered system reinforced with graded crushed stone [27], typically featuring a standard trapezoidal section. However, these subgrades are characterized by significant self-weight and extensive land occupation, leading to an increased need for foundation reinforcement, among other disadvantages. In response to these challenges, various new types of subgrade structures have been proposed and are being researched and applied in projects. One notable development in rail transit has been the use of U-shaped subgrade structures, a practice with nearly 20 years of history [28]. Ding Zhaofeng et al. [29] discussed the design of a novel U-shaped groove structure, focusing on its theoretical calculation model, internal force calculation, load analysis, and other critical technical issues, providing insights for its adoption in railway construction. KAMARUDIN N A S et al. [30, 31] conducted laboratory tests to examine the impact of varying panel sizes of U-shaped subgrade concrete slabs on soil settlement. Their findings indicated that longer web segments led to reduced settlement, highlighting the strong resistance of the slabs to lateral movement. To further explore U-shaped structures, Guo Shuaijie et al. [32–34] used a limit state design to study cantilever U-shaped subgrade structures and proposed methods for their design, optimization, and settlement analysis, focusing on load-bearing capacity, crack control, fatigue resistance, and deflection deformation.

The adoption of U-shaped subgrade structures has effectively reduced the footprint and volume of subgrade treatment. Despite these advancements, the persistent use of layered filling within U-shaped troughs has failed to overcome the fundamental shortage of high-quality fill materials. Inspired by the application of box girders, a novel solution has emerged in recent years: the concrete box subgrade. This innovative approach eliminates the requirement for traditional embankment fill materials and streamlines the construction process. As a result, it substantially lowers both the construction and maintenance expenses associated with high-speed railway embankment projects. Liu Ping [35] introduced an innovative assembly box culvert subgrade structure composed of multiple structural units. This design, while maintaining the height of the subgrade, reduces the use of fill materials and decreases lateral deformation and settlement. Zhu Weijie [36] established a concrete box subgrade calculation model to study the internal forces and deformation characteristics under different loads, analyse the natural frequency of the concrete box subgrade, and evaluate the dynamic response parameters. Yu Lei, Zhu Zhihui et al. [37–39] focused on the settlement limit issues of ballast track–concrete box subgrades. They explored the impact of foundation settlement on the dynamic and static mechanical characteristics from structural stress–deformation and train operation perspectives, determining the settlement limits under various scenarios. Finally, Liu Jinglei et al. [40] utilized numerical simulation methods to study the shoulder augmentation method for concrete box subgrades, optimizing their structural forms.

The concrete box subgrade, as a novel embankment structure, has not yet been widely adopted in practical engineering projects. Research into this type of subgrade has focused mainly on its structural configurations and settlement thresholds, with little attention given to its dynamic response under the cyclic loads imposed by passing trains. Moreover, most international high-speed railway systems employ double-line embankment structures. The most critical loading scenario in these systems occurs when trains travel in opposite directions on adjacent tracks, significantly increasing the potential for structural issues within the affected embankment segments. Therefore, understanding the dynamic behaviour of concrete box subgrades under such opposing traffic conditions is critically important.

In response to these developments, a hybrid modelling strategy has been used to explore the dynamic response of concrete box subgrades in double-line high-speed railways. This approach combines a vehicle track coupled dynamics model with a 3D track subgrade FE model to simulate the dynamic behaviour of the concrete box subgrade in a slab track system. The study examines two specific scenarios: single-line train operation and opposing double-line train operation. The focus of this analysis is to compare and understand the distributions and transmission patterns of the dynamic stress, dynamic displacement, and acceleration within the concrete box subgrade in these scenarios.

## 2 Model description

### 2.1 Modelling overview

**Fig 1:**
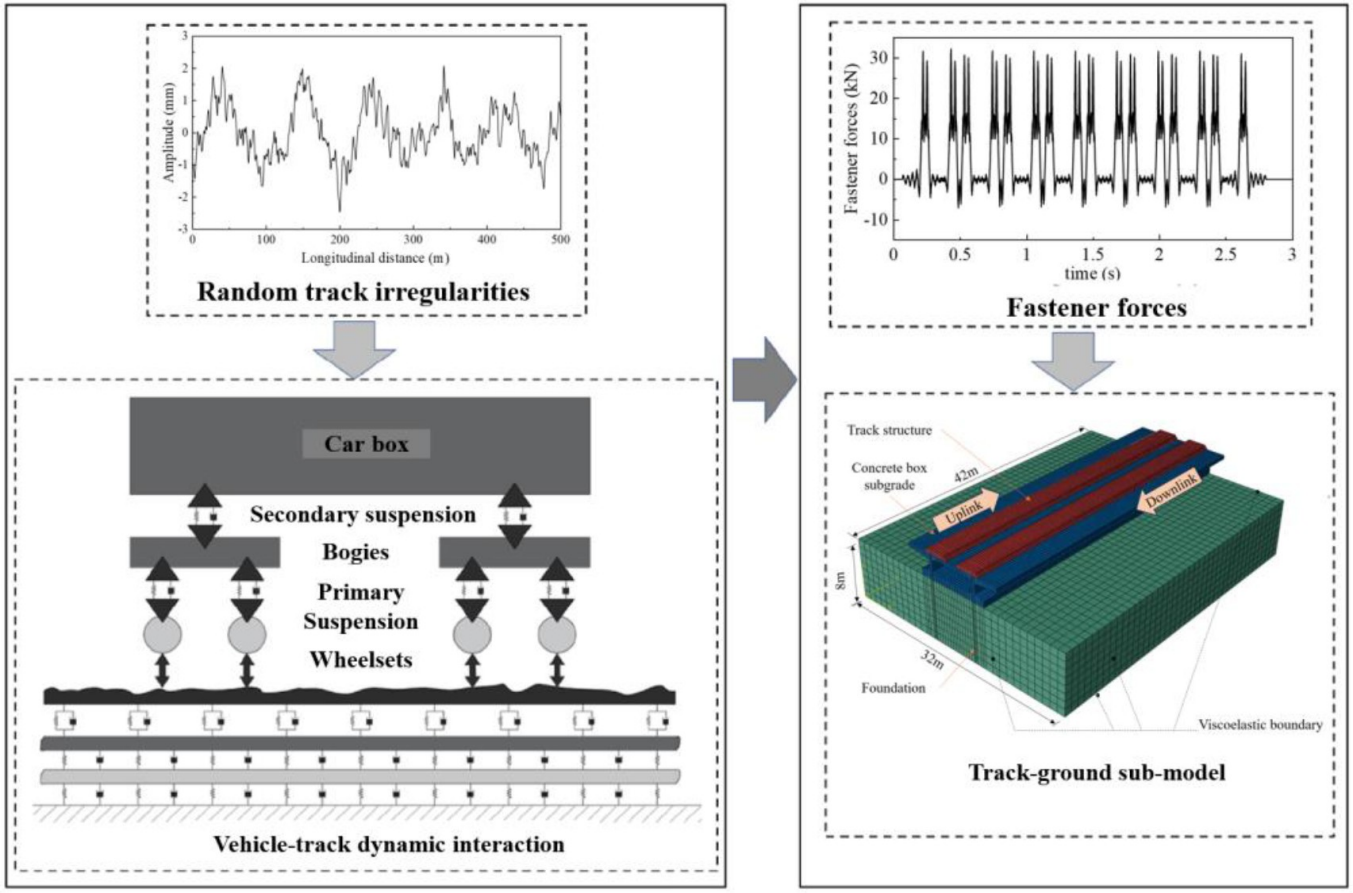


Vehicle−track vibrations arise predominantly from the irregular surfaces of wheels and rails. To analyse this interaction, a vehicle−track coupled dynamic model is employed to compute the forces on fasteners. To assess the dynamic response of different subgrades during train transit, a track−subgrade model must incorporate nonlinear material properties and intricate track geometry. A hybrid modelling strategy was proposed and executed in the time domain to fulfil these objectives. The primary modelling phases are depicted in Fig 1.

#### 2.1.1 Vehicle‒track interaction

This section employs vertical vehicle‒track coupled dynamics theory to calculate the forces exerted on fasteners. A comprehensive model is constructed to analyse the vertical interactions between railway vehicles and tracks. The moving vehicle is simulated as a multibody system with ten degrees of freedom (DOFs), while the track substructure is depicted as an infinite Euler beam. This equivalent beam is supported by an elastic foundation comprising three layers: the rail, the track slab, and the concrete base. The interaction between the vehicle and track subsystems is facilitated through wheel/rail contact, which is characterized via Hertzian nonlinear elastic contact theory. The system’s excitations are derived from random track irregularities introduced through a time−frequency transformation technique.

#### 2.1.2 Track–ground model

This component assesses the dynamic response of the track–subgrade system by employing the finite element method (FEM) in the time domain. A three-dimensional finite element (3D FE) model is developed, taking into account the nonlinear material properties and geometry of concrete box subgrades. This submodel is instrumental in calculating the dynamic stress, deflection, and acceleration of the concrete box subgrade. Nonreflective boundary conditions are implemented to ensure wave propagation towards the far-field area while preventing the reflection of outwards-propagating waves back into the domain of interest. The fastener forces derived from the vehicle‒track interaction serve as inputs for this 3D FE track−ground model.

### 2.2 Vehicle−track coupled dynamics

#### 2.2.1 Dynamic equations of motion

**Fig 2:**
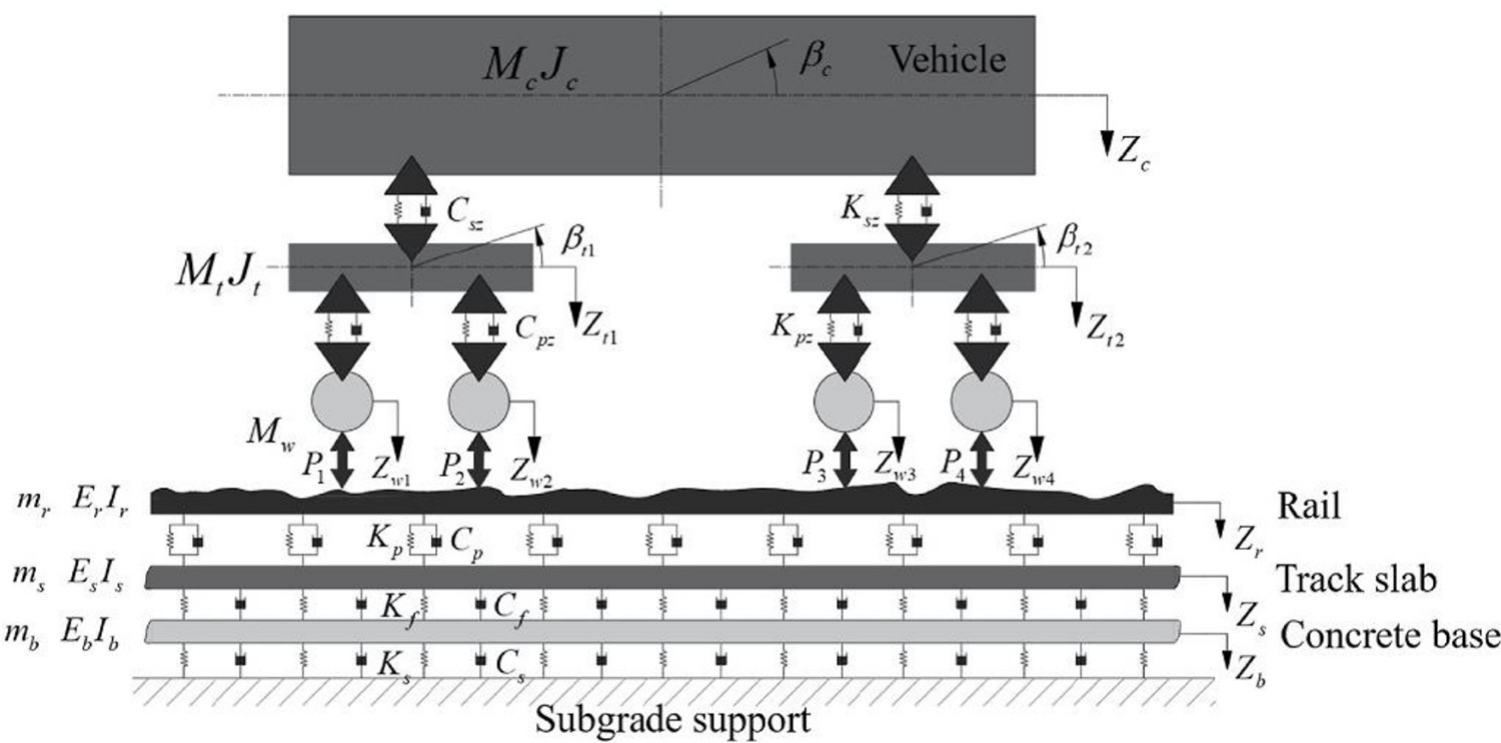


A vertical coupled dynamics model for vehicle‒track interactions, including subgrade support, is formulated to delineate the dynamic interplay among the vehicle, slab track structure, and subgrade support, as illustrated in Fig 2.

The vehicle configuration comprises a single car body, two bogie frames, and four wheelsets, which are structured within a four-axle mass−spring−damper system. Spring−damping elements, which represent the primary and secondary suspensions, facilitate the interconnection of these structural components. Both the bogie frames and the car body are attributed two DOFs, accommodating vertical and pitch movements. The wheelsets are constrained to a singular DOF, permitting solely vertical motion. The dynamic equations governing the vehicle−track interaction are formulated in submatrix notation, as detailed in references [41, 42]:

$$\left[\begin{array}{cc} M_{V} & 0\\ 0 & M_{T} \end{array}]\{\begin{array}{c} {\ddot{X}}_{V}\\ {\ddot{X}}_{T} \end{array}\}+[\begin{array}{cc} C_{V} & 0\\ 0 & C_{T} \end{array}]\{\begin{array}{c} {\dot{X}}_{V}\\ {\dot{X}}_{T} \end{array}\}+[\begin{array}{cc} K_{V} & 0\\ 0 & K_{T} \end{array}]\{\begin{array}{c} X_{V}\\ X_{T} \end{array}\}=\{\begin{array}{c} F_{V}\\ F_{T} \end{array}\right]$$ (1)

where the subscripts ’V’ and ’T’ denote the vehicle and track subsystems, respectively; [M], [K], and [C] symbolize the mass, stiffness, and damping matrices of the system, respectively; and {F} and {X} represent the force and displacement subvectors, respectively. The model incorporates nonlinear contact forces to account for wheel‒rail interactions.

The finite Bernoulli–Euler beam, which represents the rail, is supported discretely by rail fasteners. Employing the Ritz method, the dynamic equation of rail motion can be articulated as follows [41, 42]:

$$\{\begin{array}{c} {\ddot{q}}_{k}(t)+\frac{EI_{Y}}{m_{r}}(\frac{k\pi }{l})q_{k}(t)=-\sum_{i=1}^{N}F_{rsi}(t)Z_{k}(x_{i})+\sum_{j=1}^{4}p_{j}(t)Z_{k}(x_{wj}),k=1∼NM\\ Z_{r}(x,t)=\sum_{k=1}^{NM}Z_{k}(x)q_{k}(t) \end{array}$$ (2)

where the subscript ‘*k*’ pertains to the fastener; *q* (*t*) symbolizes the generalized coordinate for vertical rail motion; *EI*_*Y*_ indicates the rail’s bending stiffness relative to the Y-axis; *Z*_*r*_ (*x*, *t*) describes the rail’s vibrational displacement; *x*_*wj*_ and *x*_*i*_ denote the position coordinates of the *j*^*th*^ wheelset and *i*^*th*^ fastener point, respectively; *P* represents the wheel/rail contact force; *F*_*rs*_ is the fastener force; *N* signifies the total count of rail fasteners; and *NM* indicates the order number of the rail vibration mode.

The track slab is conceptualized as a simply supported Euler–Bernoulli beam resting on the concrete base, which is supported by a nonlinear viscoelastic subgrade. Employing the Ritz method, the dynamic motion equations for the track slab and concrete base are established as follows [41, 42]:

$$\{\begin{array}{l} {\ddot{T}}_{sn}(t)+\frac{E_{s}I_{s}L_{s}\beta_{n}^{4}}{m_{s}}T_{sn}(t)=\sum_{j=1}^{n_{0}}\frac{F_{rsj}(t)}{m_{s}}X_{n}(x_{j})-\frac{k_{f}}{m_{s}}[L_{s}T_{sn}(t)-\int_{0}^{L_{s}}X_{sn}(t)Z_{b}(x,t)dx]\\ \;-\frac{c_{f}}{m_{s}}[L_{s}{\dot{T}}_{sn}(t)-\int_{0}^{L_{s}}X_{sn}(t){\dot{Z}}_{b}(x,t)dx],n=1∼NMS\\ \;Z_{b}(x,t)=\sum_{n=1}^{NMB}X_{bn}(x)T_{bn}(t) \end{array}$$ (3)

$$\{\begin{array}{c} {\ddot{T}}_{bn}(t)+\frac{E_{b}I_{b}L_{b}\beta_{n}^{4}}{m_{b}}T_{bn}(t)=\frac{k_{f}}{m_{b}}[\int_{0}^{L_{b}}X_{bn}(t)Z_{s}(x,t)dx-L_{b}T_{bn}(t)]+\frac{c_{f}}{m_{b}}[\int_{0}^{L_{b}}X_{bn}(t){\dot{Z}}_{s}(x,t)dx-L_{b}{\dot{T}}_{bn}(t)]\\ -\frac{k_{s}}{m_{b}}L_{b}T_{sn}(t)-\frac{c_{s}}{m_{b}}L_{b}T_{sn}(t),n=1∼NMB\\ Z_{s}(x,t)=\sum_{n=1}^{NMS}X_{sn}(x)T_{sn}(t) \end{array}$$ (4)

where *X*_*n*_ (*x*) represents the mode function of the slab with coordinate *x*; *β*_*n*_ is the frequency coefficient corresponding to *X*_*n*_ (*x*); *T*_*n*_ (*t*) denotes the generalized coordinate related to time *t*; *NMS* and *NMB* indicate the mode numbers of *X*_*n*_ (*x*) for the slab and concrete base models, respectively; *Z*_*s*_ (*x*, *t*) and *Z*_*b*_ (*x*, *t*) describe the vibrational displacements of the track slab and concrete base, respectively; *E*_*s*_*I*_*s*_ and *E*_*b*_*I*_*b*_ refer to the bending stiffness of the slab and base, respectively; *m*_*s*_ and *m*_*b*_ represent the masses of the track slab and concrete base, respectively; *k*_*f*_ and *c*_*f*_ denote the stiffness and damping of the cement asphalt mortar layer between the track slab and base, respectively; *k*_*s*_ and *c*_*s*_ are the stiffness and damping of the concrete box subgrade support, respectively; *L*_*s*_ and *L*_*b*_ signify the lengths of the slab and base, respectively; and *n*_0_ is the total number of rail fasteners affixed to a single slab.

#### 2.2.2 Vertical wheel/rail interaction

The vertical wheel−rail contact force is pivotal in defining the dynamic interaction between the wheel and rail. The application of Hertzian nonlinear contact theory yields the following expression:

$$P_{wrj}(t)=\{\begin{array}{l} {\left[\frac{1}{G_{wr}}\delta Z_{j}(t)\right]}^{3/2},\;\delta Z_{j}(t)>0\\ 0,\;\delta Z_{j}(t)\leq 0 \end{array}$$ (5)

where *G*_wr_ is the constant concerning the wheel/rail contact condition and *δZ*_j_(t) is the compression at the *j*^th^ contact point. This compression is determined by accounting for vertical track irregularities alongside the displacements of both the wheel and rail.

#### 2.2.3 Random track irregularities

Vehicle‒track vibrations are predominantly induced by irregularities on the surfaces of wheels and rails. There are two principal categories of geometric irregularities: (1) specific irregularities, including wheel flats, out-of-round wheels, dipped rail joints, and rail corrugation; and (2) random irregularities, such as surface roughness on wheels and rails, which affect both the wheel–rail rolling noise and track geometry. Random track irregularity is inherent in all railway lines and is one of the factors that affects the dynamic behaviour of vehicles and tracks. Numerous factors influence specific irregularities, making them difficult to ascertain. In contrast, random track irregularities can be selected based on the standard TB/T 3352–2014. This study concentrates on the dynamic responses instigated by random track irregularities.

Random track irregularities are characterized via power spectral density (PSD) as a function of spatial frequency, for which various models exist. The approach adopted in this study follows the TB/T 3352–2014 standard issued by the National Railway Administration of the People’s Republic of China, categorizing tracks into different classes to assess track unevenness [43]. By incorporating the track spectrum into the vehicle–track system, the PSD is converted into a representation of rail geometry that varies along the track’s longitudinal profile (or in the time domain) via a time–frequency transformation technique.

### 2.3 Finite element model

A 3D dynamic FE model of a track structure was constructed via the software ABAQUS 2022 to conduct extensive analyses of the concrete box subgrade’s dynamic responses within a ballastless track system (CRTS II) subjected to train loads. The fastener forces were determined through the coupled dynamics model in the preceding computational phase and subsequently incorporated into the finite element model as excitation.

#### 2.3.1 General description

**Fig 3:**
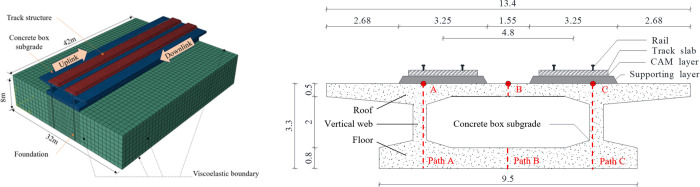


In line with TB 10621–2014 [44], the track structure features a CRTS II-type ballastless track slab consisting of a track slab, a cement asphalt mortar (CAM) layer, and a supporting layer. The concrete substructure includes a concrete box subgrade with a roof slab, vertical slab, and floor slab. The foundation soil of the model is treated as homogeneous, as illustrated in Fig 3A.

The dimensions of the model are specifically designed for this study; it spans 42 m longitudinally along the railway, with the foundation extending 32 m in width and 8 m in depth. The tracks are positioned 4.8 m apart, adhering to a standard gauge of 1.435 m, with fasteners arranged at intervals of 0.65 m. The CRTS II-type track slab is depicted with a width of 2.55 m and a thickness of 0.2 m. The CAM layer, which aligns in width with the track slab, has a thickness of 0.1 m. The supporting layer is 3.25 m wide and 0.3 m thick. C40 concrete is employed for the concrete box subgrade, and its cross-sectional details are provided in Fig 3B.

Minimal sliding is presumed between the slab track and subgrade surface, as well as between the subgrade bottom and subsoil, negating the need to account for friction between these interfaces. A tie constraint is applied to ensure coordinated deformation across the model. All model components are represented via 8-node solid elements (C3D8R). Both the track structure and the concrete box subgrade follow the linear elastic constitutive model. The subsoil is characterized by a linear elastic‒perfectly plastic model following the Mohr‒Coulomb failure criterion. The model’s calculation parameters are detailed in Table 1.

**Table 1:** Material properties of the key components of the track superstructure [45].

Components	Bulk Density (kg/m^3^)	Modulus (MPa)	Poisson’s ratio	Damping ratio	Friction angle	Cohesion (kPa)
Track slab	2500	35 500	0.167	0.03	—	—
CAM layer	2 400	8 500	0.340	0.05	—	—
Supporting layer	2 500	25 500	0.167	0.03	—	—
Concrete box subgrade	2 500	33 000	0.167	0.03	—	—
Subsoil	1 800	100	0.350	0.08	26	20

#### 2.3.2 Viscoelastic artificial boundary

In the numerical simulation, several techniques, including the boundary element method, infinite element method, and artificial boundary conditions, are employed to mitigate boundary effects. Viscoelastic artificial boundary conditions are adopted, facilitating the propagation of vibration waves towards the far-field area and inhibiting the reflection of outwardly propagating waves into the model.

Dampers and springs are affixed independently to certain boundaries in both normal and tangential orientations. The parameters for these springs and dampers are derived via Eq 6 and Eq 7.

$$K_{BN}=\alpha_{N}\frac{G}{R},C_{BN}=\rho C_{P}$$ (6)

$$K_{BT}=\alpha_{T}\frac{G}{R},C_{BT}=\rho C_{S}$$ (7)

where *K*_*BN*_ and *K*_*BT*_ denote the normal and tangential stiffness coefficients of the spring, respectively; *C*_*BN*_ and *C*_*BT*_ represent the normal and tangential damping coefficients of the dampers, respectively; and *α*_*N*_ and *α*_*T*_ are the correction coefficients in the normal and tangential directions, respectively. The recommended range for *α*_*N*_ is 1.0–2.0, whereas for *α*_*T*_, it is 1.0–2.0. For this study, *α*_*N*_ and *α*_*T*_ are set as 1.33 and 0.67, respectively [46]; *R* signifies the distance from the vibration source to the boundary; and *C*_*P*_ and *C*_*S*_ indicate the velocities of the compression and wave, respectively, which are determined via the following calculations:

$$C_{P}=\sqrt{\frac{\left(4/3G+K\right)}{\rho }}$$ (8)

$$C_{S}=\sqrt{\frac{G}{\rho }}$$ (9)

where *G* represents the shear modulus; *K* denotes the bulk modulus; and *ρ* is the mass density of the structure layer.

#### 2.3.3 Train loading

The simulation depicts the operation of a CRH380 train moving on a double-line high-speed railway. This configuration includes an 8-car formation, with each car bearing an axle load of 17 tonnes, and operates at a velocity of 300 km/h. The specific calculation parameters for the training set are detailed in Table 2.

**Table 2:** Calculation parameters for the CRH380 vehicle.

Parameters	Symbols	Units	Values
Body mass	*M*_c_	kg	42,934
Body nodding inertia	*J*_c_	kg∙m^2^	1.7118×10^6^
Bogie quality	*M*_t_	kg	3,300
The bogie nods its moment of inertia	*J*_t_	kg∙m^2^	1,807
Wheelset mass	*M*_w_	kg	1,780
Wheelset nodding inertia	*J*_w_	kg∙m^2^	1180
Suspension stiffness of a series	*K*_pz_	N∙m^-1^	1.176×10^5^
First suspension damping	*C*_pz_	N∙s∙m^-1^	1.0×10^4^
Secondary suspension stiffness	*K*_sz_	N∙m^-1^	2.4×10^5^
Secondary suspension damping	*C*_sz_	N∙s∙m^-1^	2.0×10^4^
Spacing of bogies in the same car	*L*_c_	m	17.5
Same bogie wheelbase	*L*_t_	m	2.5
Train length	*L*	m	25

**Fig 4:**
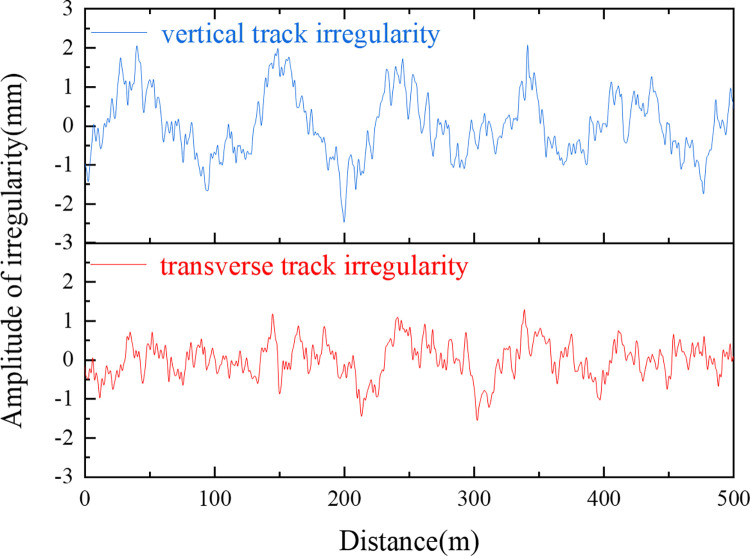


The track irregularity spectrum, which is essential for this simulation, is derived via vehicle-coupling dynamics [42] and the high-speed railway spectrum [43]. This spectrum, illustrated in Fig 4, is obtained via inverse Fourier transformation and acts as an external excitation within the wheel‒rail interaction model.

**Fig 5:**
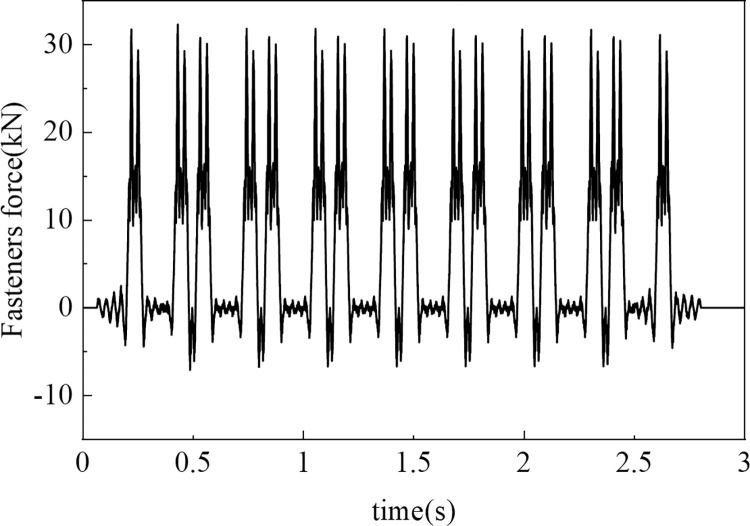


The vehicle‒track coupled dynamics yield a series of curves of fastener forces vs. time. These curves represent the dynamic loads exerted by the train on the track and are critical for the accuracy of the simulation. The loads are applied at the respective fastener locations on the track slab, thereby precisely emulating the influence of train loads on the track structure. A schematic representation of the fastener loads and their application on the track slab is provided in Fig 5.

## 3 Validation

Concrete box subgrades represent an emerging concept within subgrade structures. To date, there has been limited exploration in both theoretical and empirical research. To bridge this gap, the present study adopts a comparative approach. Drawing on measured field data from the conventional subgrade of the Wuhan−Guangzhou high-speed railway [47], the results derived from both conventional and concrete box subgrade models are compared. The conventional subgrades are established via uniform computational parameters, as outlined in Table 3. This comparative analysis indirectly substantiates the accuracy and reliability of the computational model employed in this investigation.

**Table 3:** Material properties of the key components of the track superstructure [47].

Components	Bulk Density (kg/m^3^)	Modulus (MPa)	Poisson’s ratio
Track slab	2 500	35 000	0.17
CAM layer	1 800	95	0.40
Supporting layer	2 500	24 000	0.20
Concrete box subgrade	2 500	33 000	0.167
Subsoil	2 000	600	0.35

**Fig 6:**
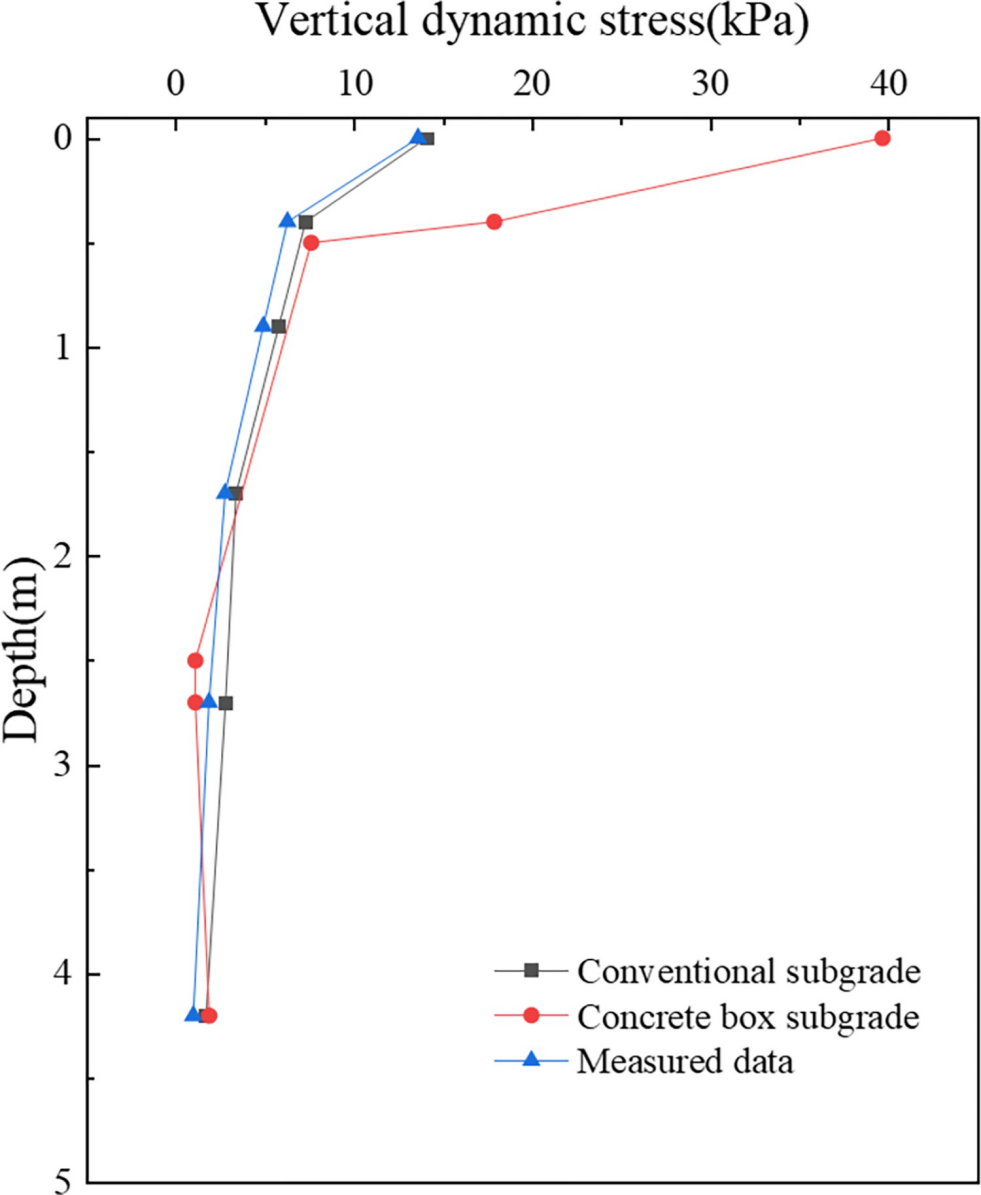


Fig 6 presents a comparison of the vertical dynamic stresses within the subgrade, illustrating the dynamic stress distribution along the depth. The results of the conventional subgrade from this study demonstrate a high degree of congruence with the measured data, as depicted in Fig 6. Although the dynamic stresses calculated for the conventional subgrade slightly exceed the measured values, with peak stresses recorded at 13.6 kPa and 14.0 kPa, the variance remains within 2.9%. In the case of the concrete box subgrade, despite a more pronounced difference at the surface of the box roof, the overall distribution closely mirrors the measured data. This variation is attributable to the material properties of the concrete box subgrade, which tend to exhibit stress concentrations at the box roof surface, whereas this disparity diminishes with increasing depth.

Field-measured data from the Wuhan−Guangzhou railway line show that the dynamic displacement on the subgrade surface ranges from 0.003 mm to 0.211 mm [47]. The dynamic displacements calculated for both conventional and concrete box subgrades in this research fall within this observed range, registering at 0.19 mm and 0.12 mm, respectively. The outcomes pertaining to the dynamic stress and displacement corroborate the validity and precision of the finite element model devised for this study, thus providing a reliable basis for further investigations of concrete box subgrade structures.

## 4 Results and analysis

Utilizing the corroborated modelling approach, the dynamic responses of the concrete box subgrade were assessed under two distinct scenarios: single-line operation (only uplink) and opposing double-line operation. In scenarios featuring a CRH380 vehicle running on the CRTS II slab track system, the model incorporates a train velocity of 300 km/h and an axle load of 170 kN. To comprehensively analyse the distribution and transmission of the dynamic stress, displacement, and acceleration within the concrete box subgrade, three specific positions were selected. Point A is positioned beneath the left rail of the uplink, Point B lies directly underneath the centre of the subgrade, and Point C is situated beneath the right rail of the downlink, as depicted in Fig 3.

### 4.1 Dynamic stress

#### 4.1.1 Time‒history curve

**Fig 7:**
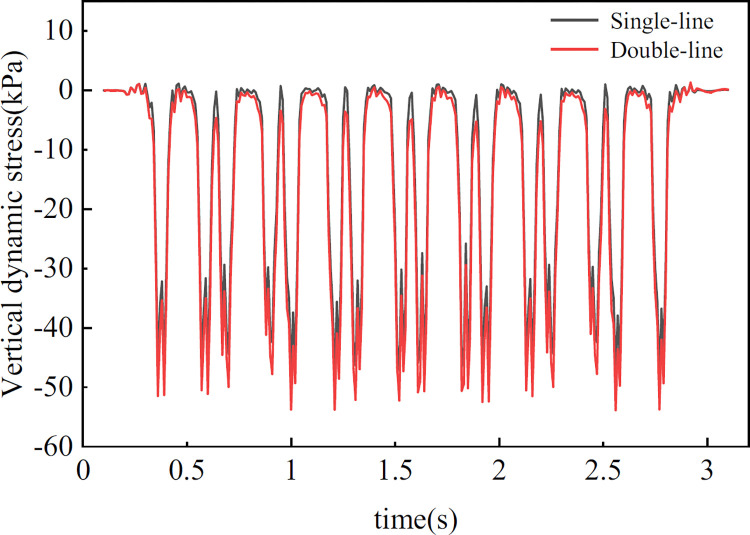


Fig 7 presents the time–history curve of the vertical dynamic stresses on the subgrade surface located at position A under single-line and opposing double-line operation. Influenced by the train load, the curve exhibits distinct periodic fluctuations, forming a ’camel hump’ pattern with 16 peak groups. These peaks correspond to the number of bogies in the 8-car train formation, with each peak representing an individual bogie. This result suggested that two superimposed axle loads of the same bogie achieve a complete loading–unloading cycle.

At a train speed of 300 km/h, the peak vertical dynamic stress recorded under single-line operation reaches 48.2 kPa, which increases to 53.9 kPa under opposite double-line operation; this indicates that the peak dynamic stress during double-line operation is 5.7 kPa greater than that during single-line operation, representing an increase of 11.8%. The impact of double-line train loads on the subgrade surface extends beyond simple linear superposition, entailing a more intricate process of stress redistribution, particularly in areas where lines intersect.

#### 4.1.2 Transverse distribution

**Fig 8:**
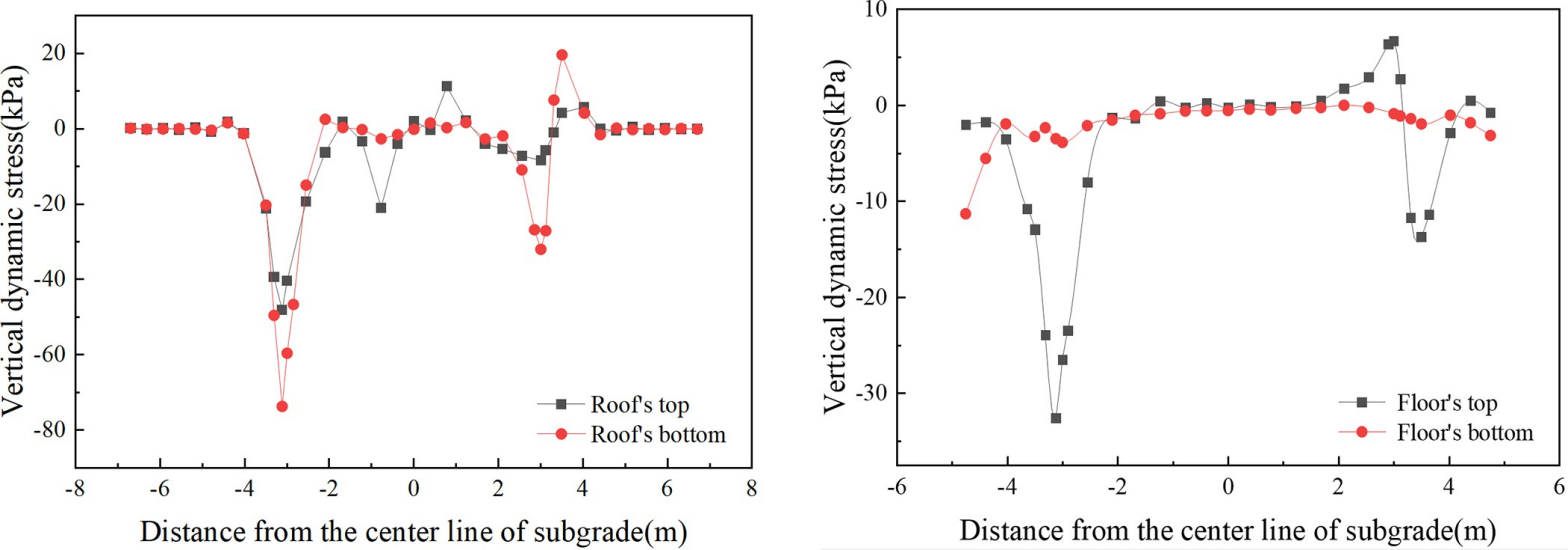


Fig 8 provides insight into the transverse distribution of vertical dynamic stress for a box subgrade roof and floor in single-line operation. In single-line operation, vertical dynamic stress responses are primarily localized within the operational line’s range, exhibiting markedly lower stress levels on the alternate track. As illustrated in Fig 8A, the box roof top surface exhibited two pronounced stress peaks at the support layer edge (-0.775 m) and at the junction between the box roof and the vertical web (-3.118 m), with stress values of 21.0 kPa and 48.2 kPa, respectively. Notably, the stress concentration at the junction between the box roof and the vertical web is more pronounced than that at the support layer edge. Similarly, on the bottom surface of the box roof, stress peaks of 73.7 kPa and 32 kPa are observed at the corresponding junctions with the vertical web. The box floor’s top surface, as depicted in Fig 8B, shows stress concentration solely at the junctions with the vertical web. Conversely, the dynamic stress on the bottom surface of the box floor remains uniform, with minimal fluctuations and a slight elevation at the plate edges, not surpassing 10 kPa.

**Fig 9:**
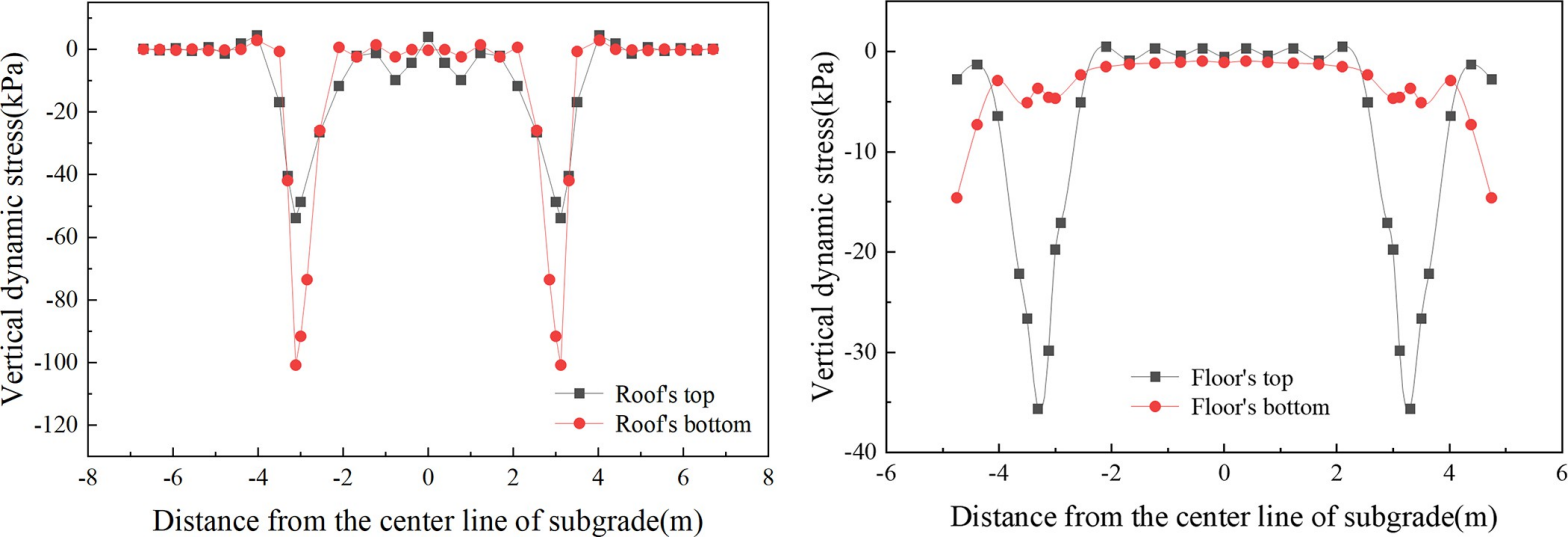


Fig 9 displays the transverse distribution of the vertical dynamic stress for both the box roof and the floor of the subgrade during bidirectional operation on parallel tracks. As depicted in Fig 9A, the vertical dynamic stress on the box roof of the subgrade exhibits a ’saddle-shaped’ distribution during bidirectional operation. The stress on both the top and bottom of the box roof increases progressively when moving away from the subgrade center line. Peaks near 3.118 m register at 53.9 kPa and 100.8 kPa, respectively. These peaks are observed at the junctions of the box roof and floor with the vertical slab, indicating more pronounced stress concentrations in these areas. In Fig 9B, the dynamic stress pattern on the top surface of the box floor mirrors that of the top surface of the box roof, revealing a ’saddle-shaped’ distribution. The peak stress here, occurring near 3.3 m, is 35.7 kPa. This peak corresponds to the junction between the vertical slabs and box floor, a region where significant stress concentration is evident. The dynamic stress observed on the bottom surface of the box floor during bidirectional operation follows a similar pattern to that observed during single-line operation but with a more symmetrical distribution.

When the dynamics of single-line and double-line operations are compared, a difference of 5.7 kPa (11.7%) in the maximum dynamic stress experienced by the subgrade surface is observed. With respect to the vertical dynamic stress distribution in the transverse direction, there is a distinct contrast between single-line and double-line operations. Under single-line operation, the distribution is uneven, with stress primarily concentrated within the range of the operational side. In contrast, during double-line operation with trains moving in opposing directions, the lateral distribution of vertical dynamic stress extends across both tracks, with stress peaks occurring at the junctions between the box roof and the vertical web on the box subgrade surface.

#### 4.1.3 Distribution along the depth

**Fig 10:**
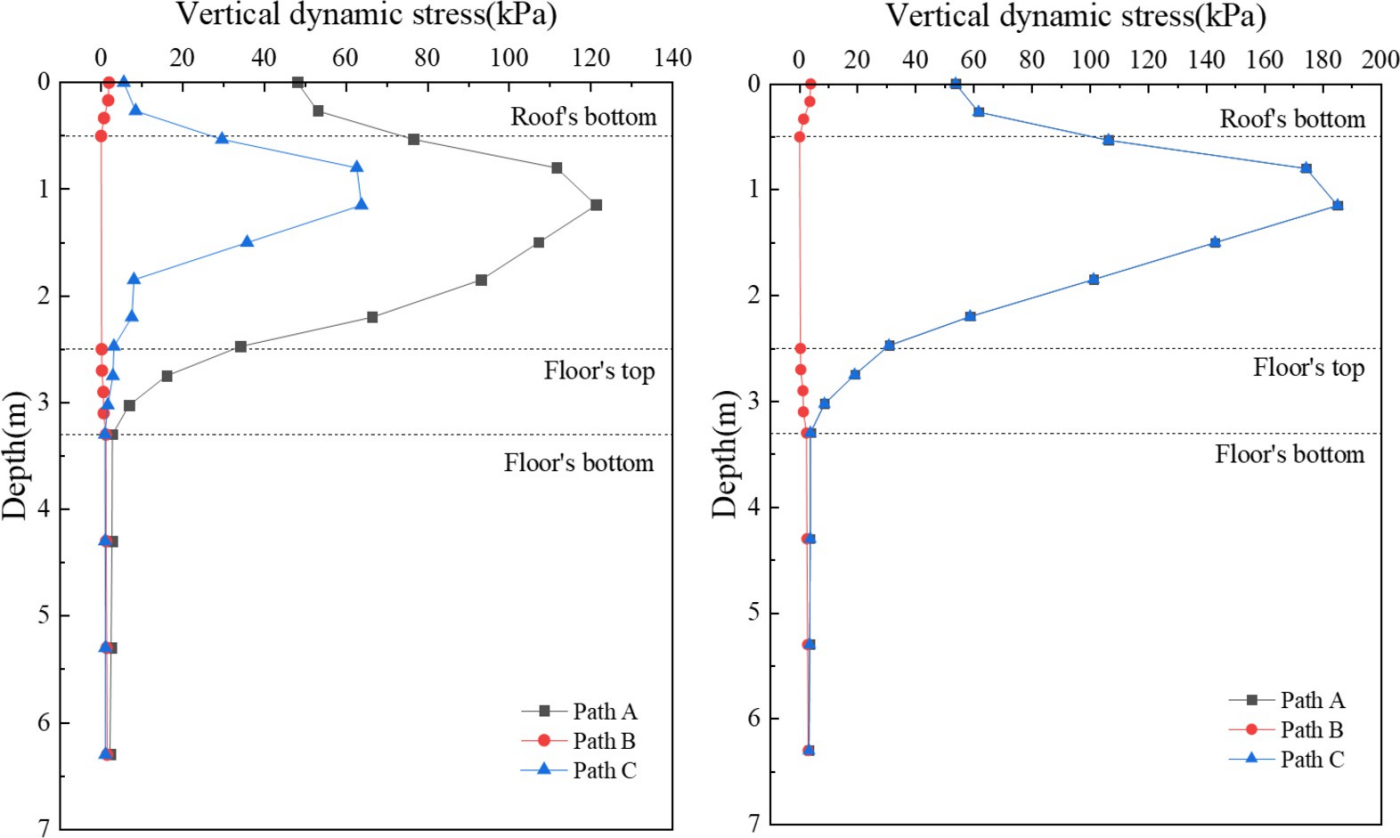


Fig 10 shows the vertical dynamic stress distribution along the depth of the concrete box subgrade. During single-line operation, as shown in Fig 10A, Points A and C on the surface of the box roof exhibit vertical dynamic stresses of 48.2 kPa and 5.7 kPa, respectively. Within the depth range of the box roof, the stress swiftly increases, peaking at the junctions where the vertical webs meet the roof’s bottom surface, with 121.3 kPa and 63.9 kPa at positions A and C, respectively. As the stress extends into the vertical web and box floor, it rapidly attenuates, decreasing to 2.9 kPa and 1.1 kPa at the underside of the box floor, respectively. At Point B on the surface of the box roof, the vertical dynamic stress is 2.0 kPa, which rapidly diminishes within the box roof. There is a slight increase within the box floor, which is attributable to reflection phenomena at the interface with the subsoil. Below a depth of 3.3 m, the dynamic stress variations across different paths become nearly identical because of the absorption and redistribution by the concrete box subgrade, minimizing the differences.

In the scenario of bidirectional operation, depicted in Fig 10B, the overall trend of the vertical dynamic stress distribution with depth mirrors that observed during single-line operation. The stress values at Points A and C on the surface of the box roof are uniform at 53.9 kPa. At the junctions with the vertical web, the stress peaks at 185.2 kPa. The stress then decreases to 3.9 kPa at the underside of the box floor. At Point B on the top surface of the roof, the vertical dynamic stress is 4.0 kPa.

**Fig 11:**
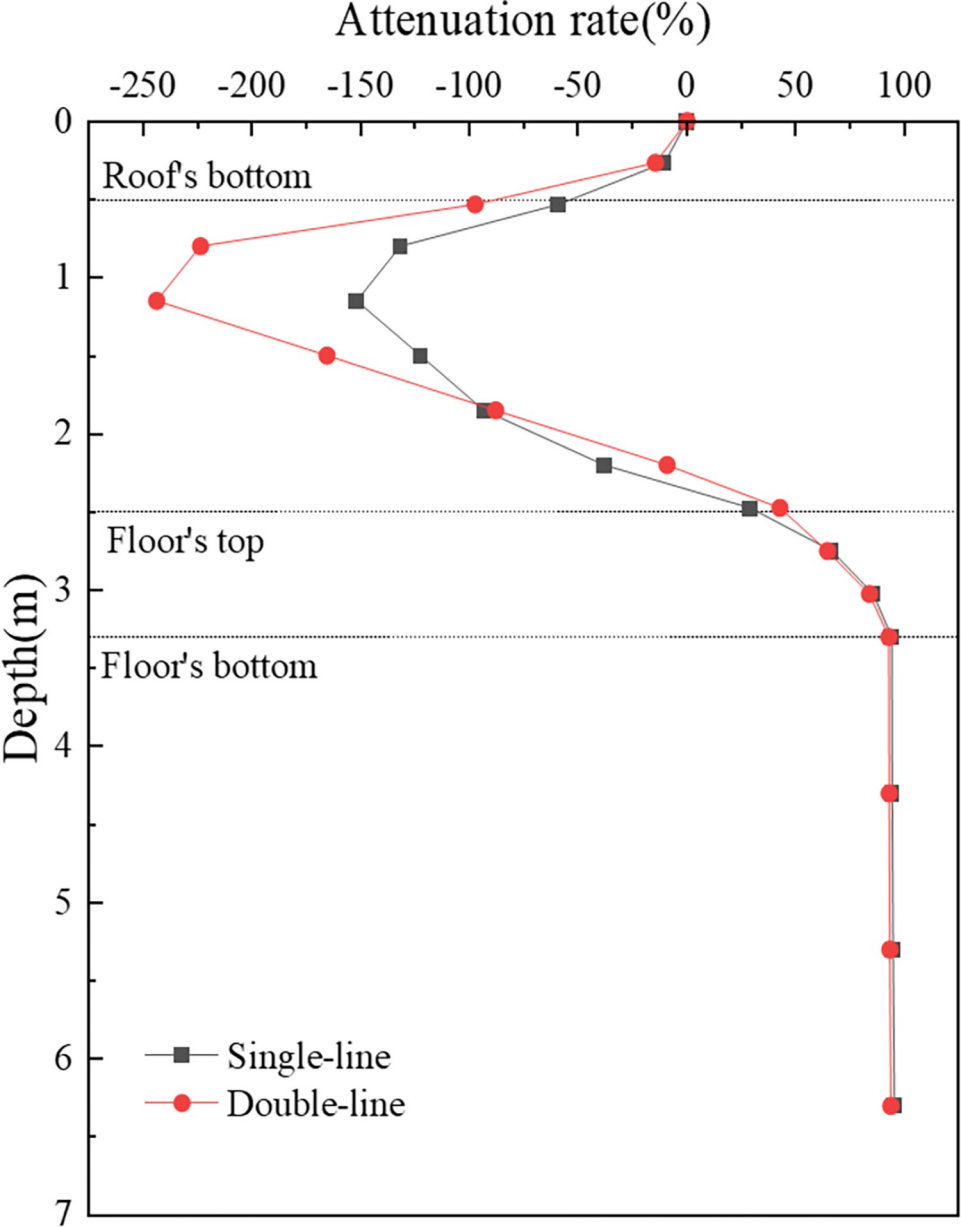


Fig 11 provides a detailed illustration of the attenuation pattern of the vertical dynamic stress amplitude, focusing on the distribution along the depth of the subgrade, specifically beneath the left rail of the uplink during both single- and double-line operations. The attenuation rate curves for both modes of operation exhibit similar trends. Within the roof of the concrete box subgrade, the attenuation rate in the reverse direction increases significantly, reaching -59.1% for single-line operations and -97.5% for double-line operations at the underside of the roof slab. In the vertical web, the vertical dynamic stress rapidly diminishes, with attenuation rates of 29% and 42.6% at the top surface of the box floor for single- and double-line operations, respectively. The attenuation continues within the box floor, culminating in rates of 94.1% and 92.7% at the underside of the floor for single- and double-line operations, respectively. These figures demonstrate the efficacy of the concrete box subgrade in absorbing and dissipating dynamic loads.

A comparison of the patterns of the vertical dynamic stress distributions during single-line and double-line operations reveals that the vertical dynamic stress under bidirectional operation is consistently greater than that under single-line operation. Bidirectional operation results in a cumulative effect on the vertical dynamic stress, which becomes more pronounced as depth increases; this underscores the importance of considering operational modes in the design and analysis of box subgrades, particularly in contexts where different traffic operations are expected.

### 4.2 Dynamic displacement

#### 4.2.1 Time‒history curve

**Fig 12:**
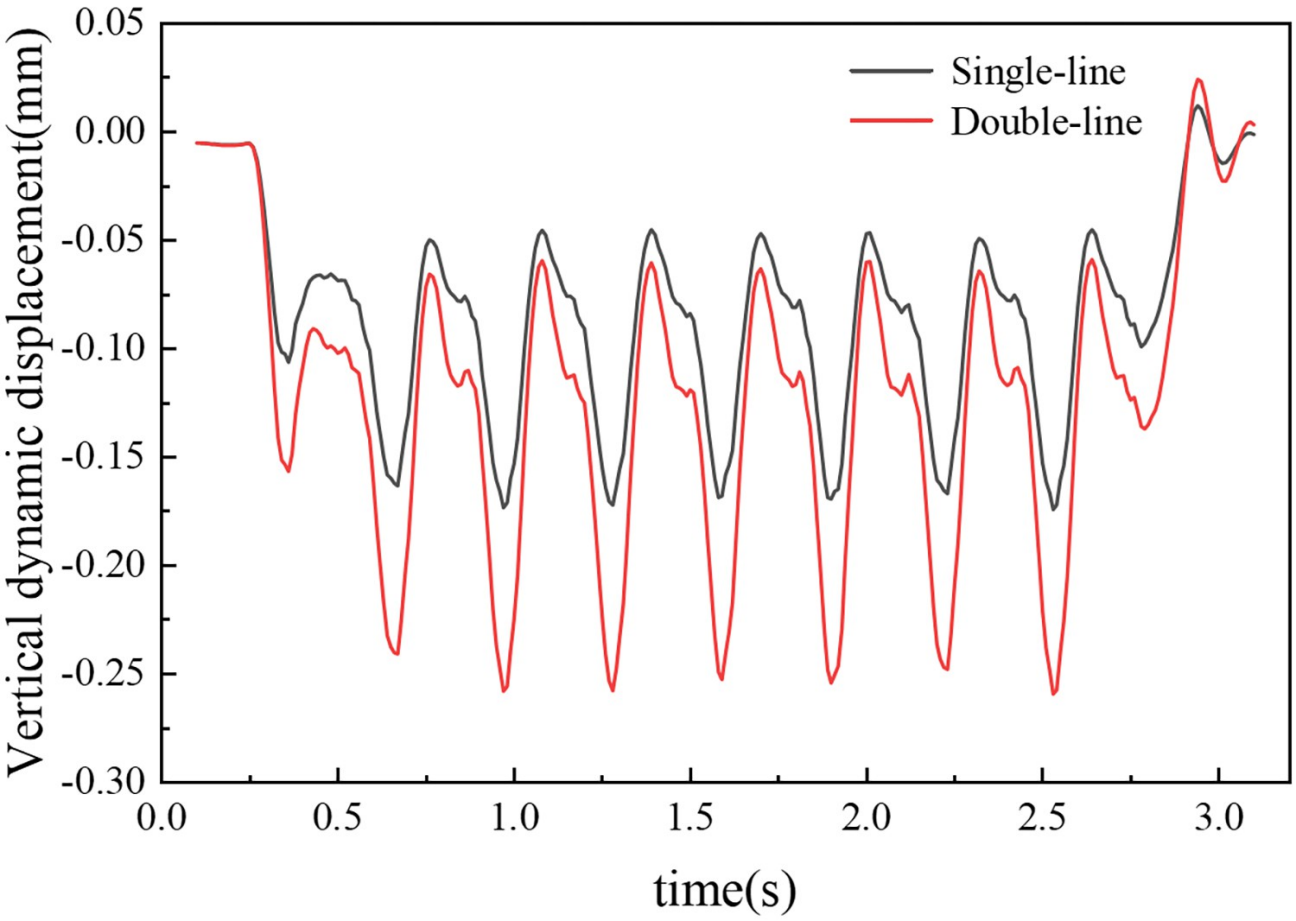


Fig 12 displays the time–history curve of the dynamic displacement on the subgrade surface at position A, which is located beneath the left rail of the upper track. The curve exhibits a ’wave-like’ distribution with nine distinct wave peaks, reflecting the passage of the train across the monitoring point. The initial group of peaks corresponds to the passage of the train’s first bogie; the middle seven peaks emerge due to the combined effect of adjacent bogies from different carriages; and the final group of peaks is a result of the train’s last bogie passing over the point. The vertical dynamic displacement sharply decreases and approaches zero as the last bogie moves away from the monitoring point.

At a train speed of 300 km/h, both single-line and bidirectional operations display consistent patterns in the phase of vertical displacement extremes. The maximum vertical dynamic displacement observed during single-line operation is 0.174 mm, whereas in bidirectional operation, it increases to 0.259 mm, approximately 1.49 times greater than that of single-line operation. This difference in vertical dynamic displacement between the two operational modes is significant. In bidirectional operation, the vertical dynamic displacement is greater than that in single-line operation, underscoring the varied impacts of operational modes on the dynamic behaviour of railway subgrades.

#### 4.2.2 Transverse distribution

**Fig 13:**
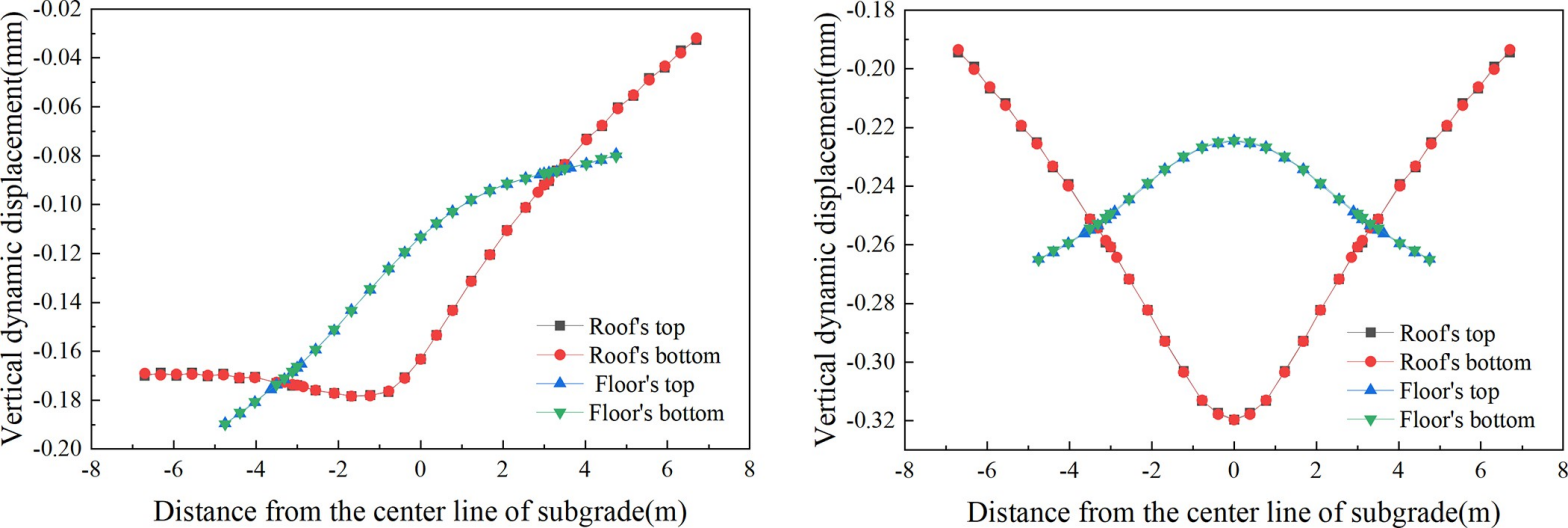


Fig 13 presents the transverse distribution of the dynamic displacement for the concrete box subgrade, revealing notable patterns in how displacement is distributed across the box subgrade under different operational conditions.

In single-line operation, where the subgrade experiences eccentric dynamic loads, there is a concentration of vertical dynamic displacement within the operational track range, predominantly on the uplink side. A relatively small displacement is observed on the downlink side. Within the box roof, the maximum displacement of 0.178 mm occurs near the right rail of the uplink. This displacement gradually decreases towards both sides. To the left, the displacement decreases slowly, reaching a minimum of 0.169 mm at the left edge of the box roof, corresponding to a maximum difference of 0.009 mm. To the right, the displacement decreases more rapidly, falling to 0.032 mm at the right edge, with a maximum difference of 0.146 mm. In the box floor, the largest vertical dynamic displacement, 0.190 mm, is found at the left edge, decreasing continuously towards the right, reaching 0.08 mm at the right edge.

During bidirectional operation, the vertical dynamic displacement displays a more symmetric distribution. Within the box roof, the maximum displacement at the centre of the subgrade is 0.320 mm, which decreases gradually towards the edges to form a "U" shaped distribution. The displacement decreases to 0.193 mm at the roof edges, a difference of 0.127 mm. In the box floor, the smallest displacement, 0.224 mm, occurs at the subgrade centre, increasing gradually towards both edges to form an inverted "U" shaped distribution. The displacement reaches 0.265 mm at the edges, with a maximum difference of 0.041 mm.

When single-line and double-line operations are compared, the maximum vertical dynamic displacement on the surface of the single-line subgrade is 0.178 mm. For the double-line operation, this value increases to 0.320 mm, approximately 1.8 times that of the single-line operation. In single-line operation, the vertical dynamic displacement is primarily concentrated within the range of the operational track. Conversely, in bidirectional operation, the displacement is fully distributed across the width of the subgrade, reflecting the differing impacts of train operation modes on the dynamic behaviour of the subgrade.

#### 4.2.3 Distribution along depth

**Fig 14:**
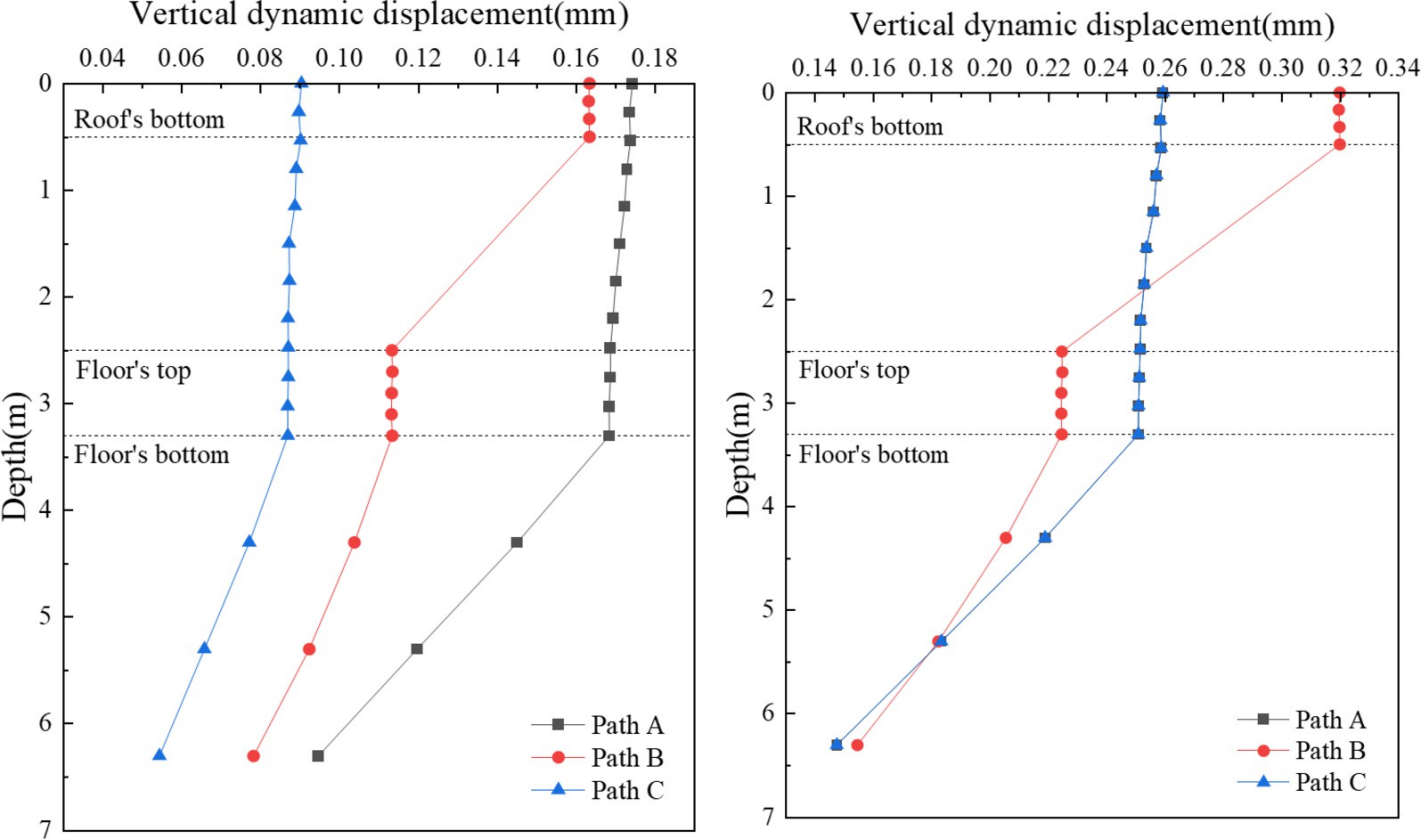


Fig 14 provides a detailed examination of the depth distribution of vertical dynamic displacement within the box subgrade, focusing on three specific paths: A, B, and C.

In single-line operation, the greatest dynamic displacement at the top surface of the roof slab is noted at Point A (0.174 mm), followed by Point B (0.163 mm) and Point C (0.090 mm), following the order A > B > C. During bidirectional operation, the largest displacement at the top surface of the roof slab is observed at Point B (0.320 mm), whereas Points A and C show nearly identical displacements of 0.259 mm each. The robust reinforced concrete structure of the concrete box subgrade results in almost equal dynamic displacements at the upper and lower surfaces of the box roof and floor. As the depth increases, the disparity in the dynamic displacement along the three paths diminishes, a trend attributed to the subgrade’s ability to absorb and redistribute stress.

**Fig 15:**
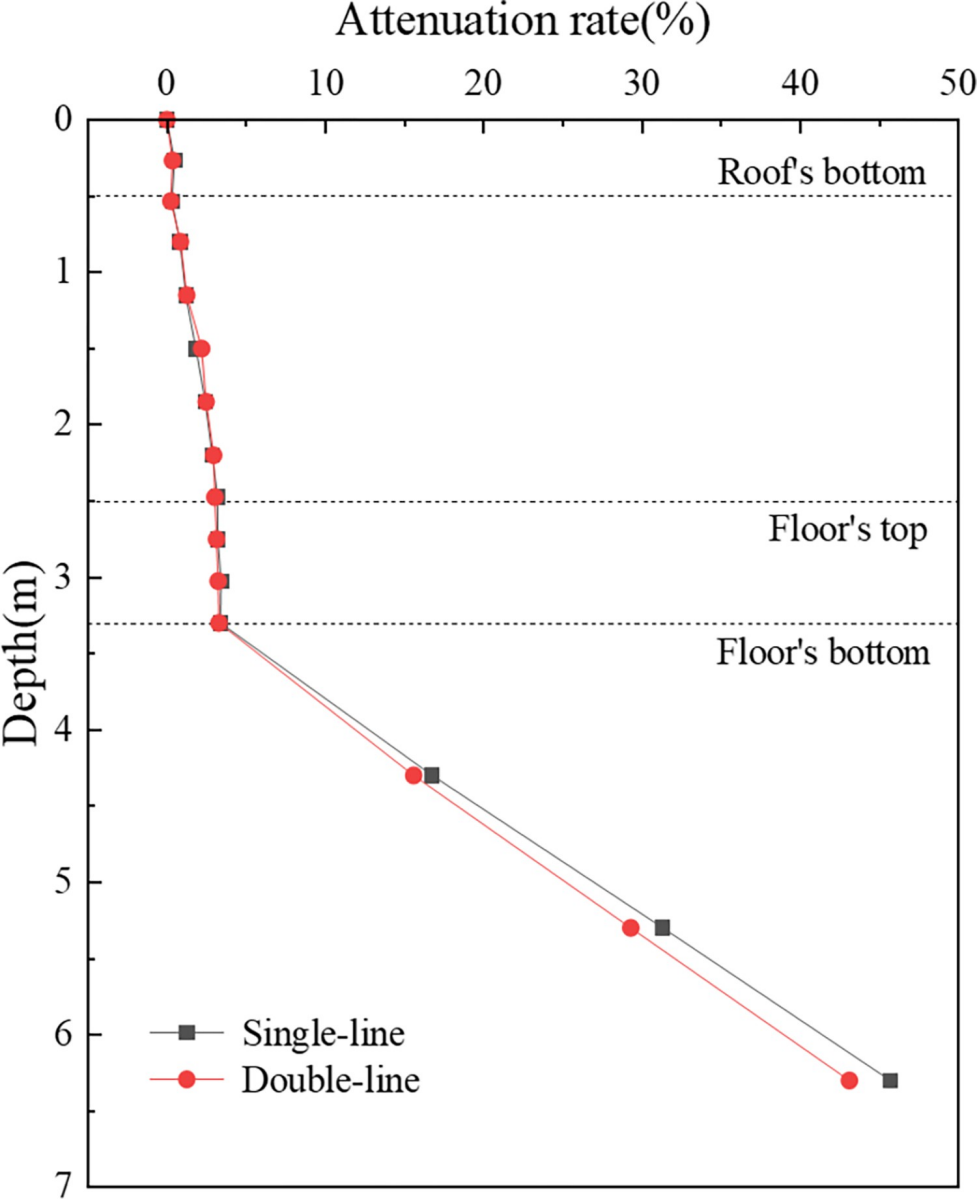


Fig 15 is utilized to more clearly delineate the attenuation pattern of vertical dynamic displacement across different operational modes. The attenuation patterns in both operational modes are broadly similar, featuring maximum displacement at the box roof surface and decreasing almost linearly with depth. Within the concrete box subgrade, the attenuation rate gradually increases, reaching 3.4% and 3.3% at the underside of the box floor for single- and double-track operations, respectively. In the foundation soil, the attenuation rate increases sharply, reaching 45.7% and 43.1% at a depth of 6 m below the subgrade surface. This suggests that the deformation caused by the concrete box subgrade is minimal, with most of the displacement originating from the foundation soil.

When single-line and double-line operations are compared, it is apparent that bidirectional operations result in a significant cumulative effect on vertical dynamic displacement, resulting in notably larger displacements than single-line operation. However, interestingly, at the same depth, the attenuation rate during bidirectional operation is slightly lower than that during single-line operation. This comparison highlights the impact of operational modes on the dynamic behaviour of railway subgrades, particularly in terms of vertical dynamic displacement and its attenuation with depth.

### 4.3 Acceleration

#### 4.3.1 Time‒history curve

**Fig 16:**
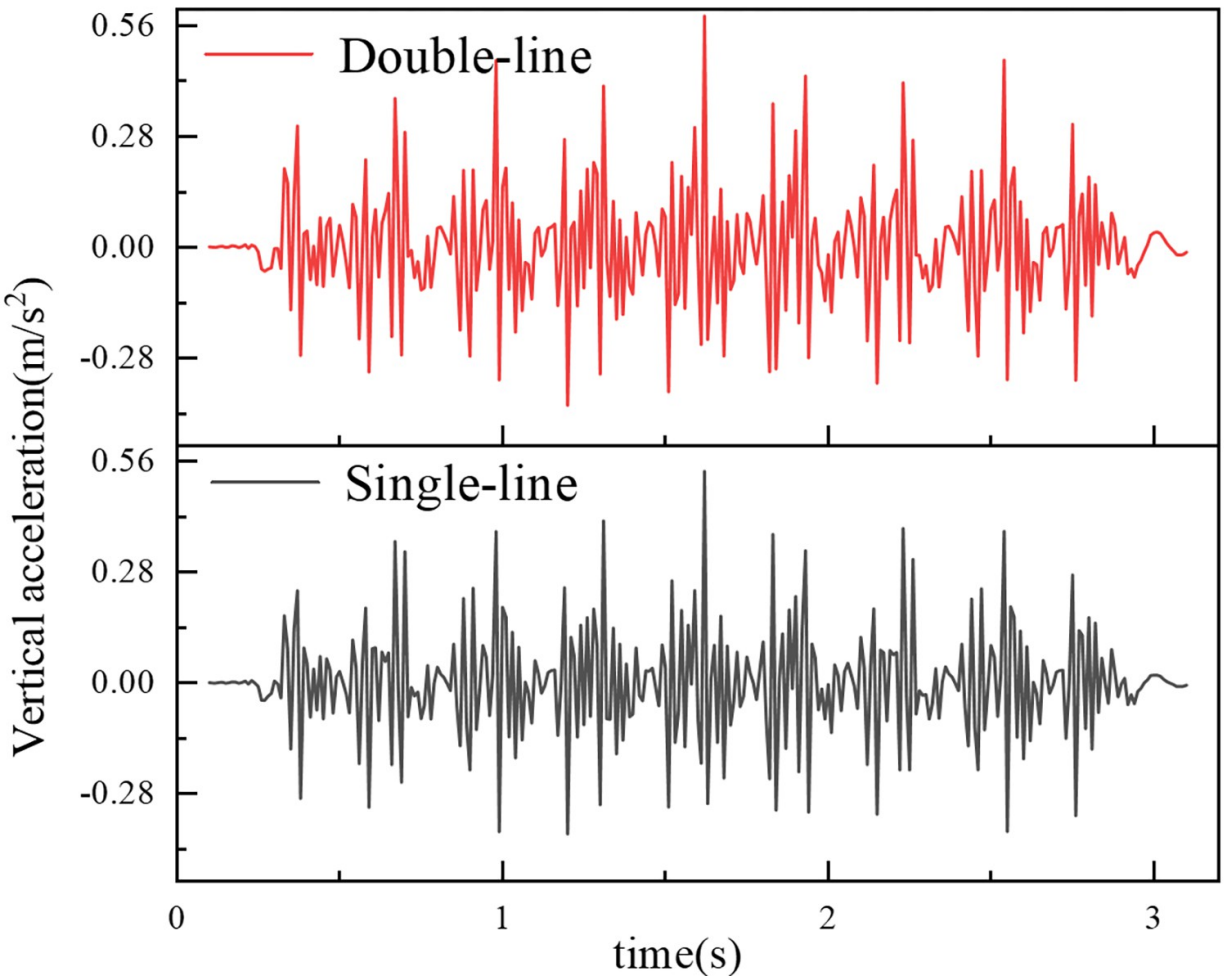


Fig 16 shows the time–history curve of the vertical acceleration at the surface of the subgrade beneath the left rail of the upper track on a concrete box subgrade. As depicted in Fig 16, the acceleration time–history curve, influenced by train loading, exhibits significant periodic variations. This pattern mirrors that observed in the dynamic stress time‒history curve, reflecting the consistent nature of these dynamic responses.

When the train operates at a speed of 300 km/h, there is a remarkable consistency in the phase of vertical acceleration extremes between single-line and bidirectional operations. During single-line operation, the maximum vertical acceleration is measured at 0.533 m/s^2^. In contrast, during bidirectional operation, this value slightly increases to 0.582 m/s^2^, a difference of only 0.049 m/s^2^. This finding indicates that the vertical acceleration experienced during bidirectional operation is somewhat greater than that experienced during single-line operation.

#### 4.3.2 Distribution along the depth

**Fig 17:**
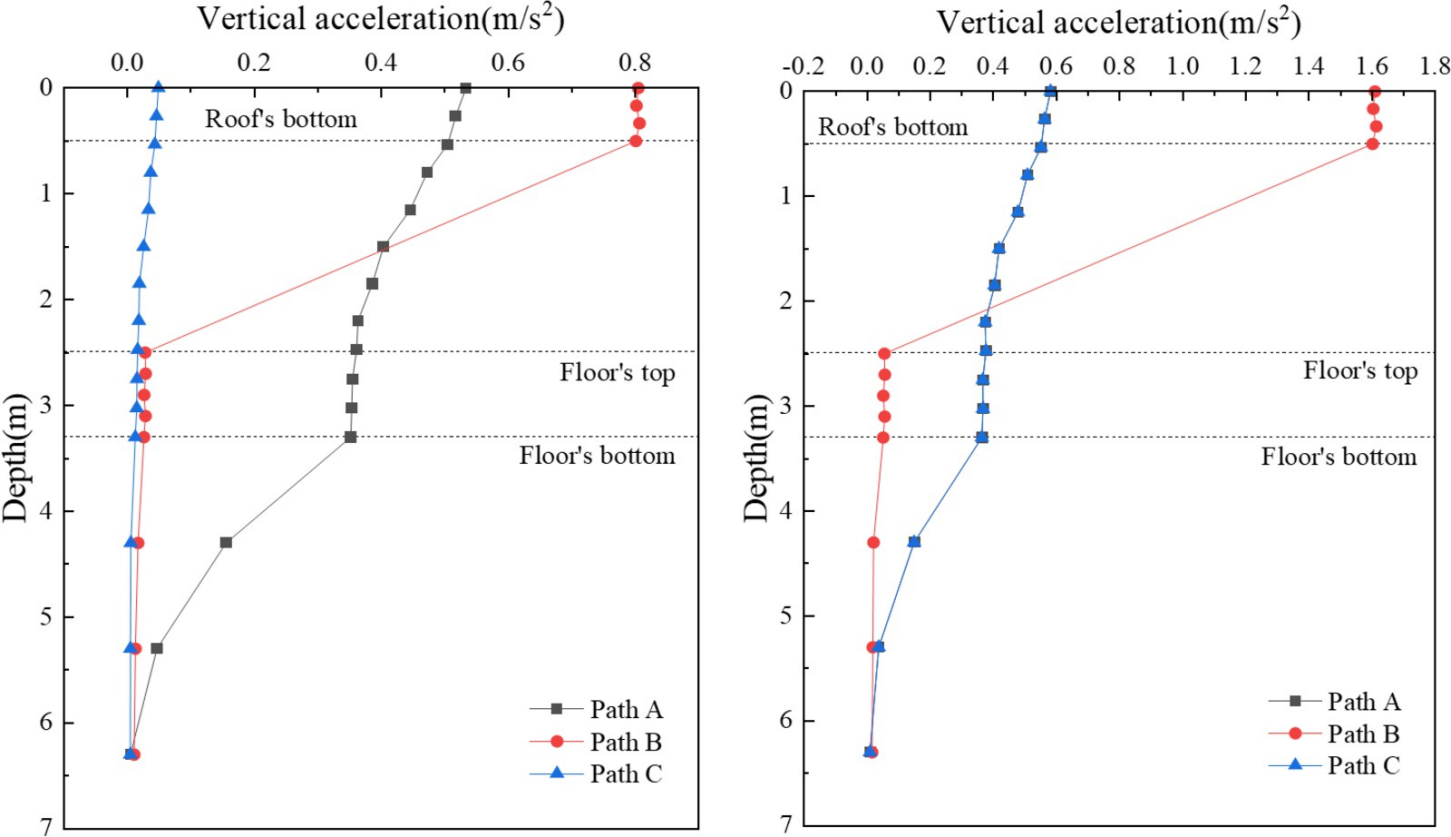


Fig 17 displays the depth distribution curves of the vertical acceleration for the concrete box subgrade. These distribution patterns are somewhat similar across all paths, with the highest acceleration noted at the surface of the box roof, which then progressively decreases with increasing depth. During single-line operation, the most pronounced acceleration at the box roof surface is observed at Point B (0.805 m/s^2^), followed by Points A (0.533 m/s^2^) and C (0.049 m/s^2^). In bidirectional operation, the highest acceleration at the roof slab surface is at Point B (1.609 m/s^2^), with Points A and C displaying nearly identical accelerations of 0.582 m/s^2^ each. As the depth increases, the variation in acceleration along the three paths becomes less significant, tending towards zero.

**Fig 18:**
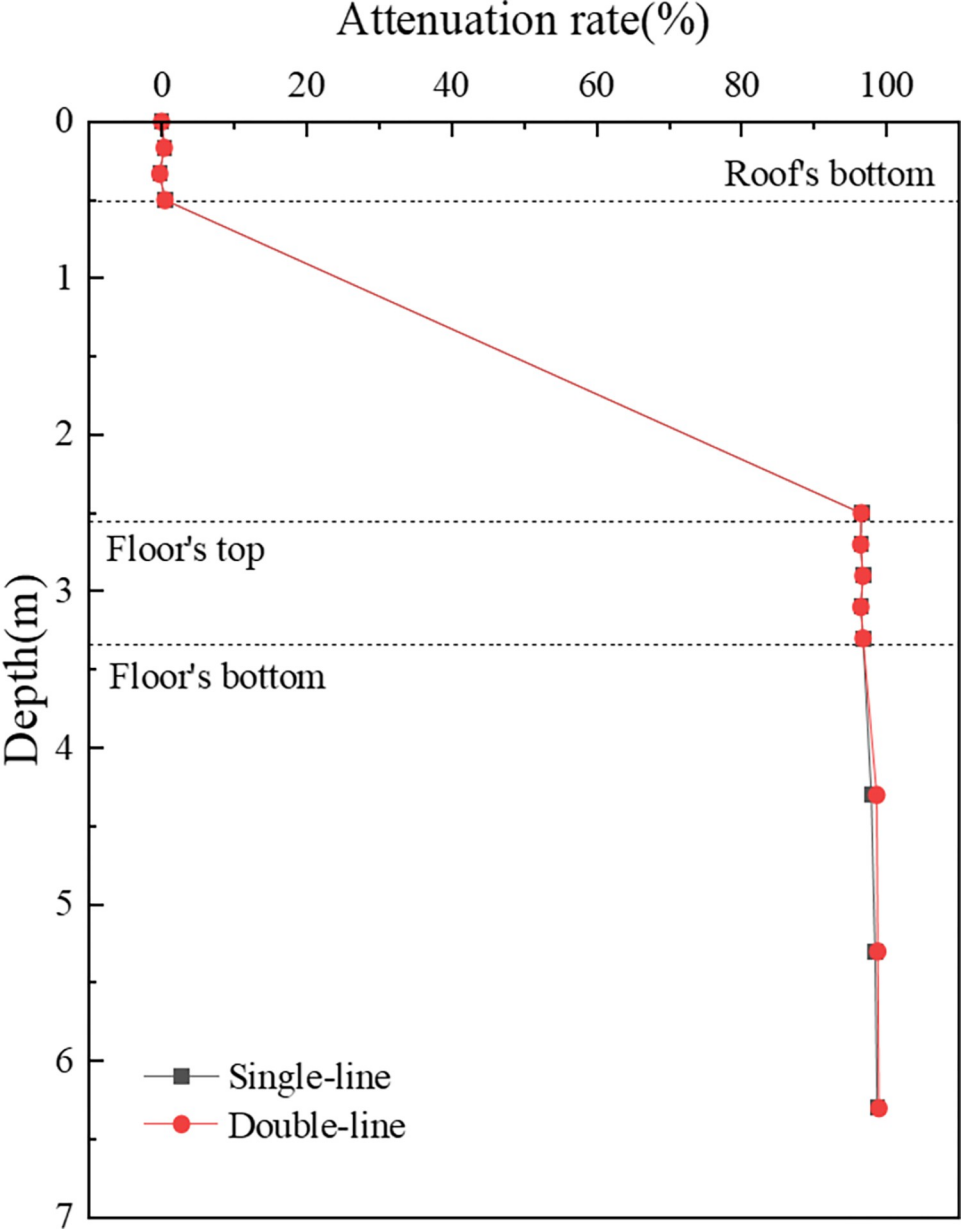


Fig 18 is used to elucidate the attenuation pattern of vertical acceleration within the concrete box subgrade. It features a decay rate curve along the path beneath the subgrade centre. The attenuation patterns for both operational modes are strikingly similar, with the maximum acceleration at the box roof surface decreasing gradually with depth. Within the box roof and floor, the acceleration shows minimal attenuation, but it decays sharply from the roof to the floor. At the bottom surface of the floor, the attenuation rates are 96.7% for single track operations and 96.8% for double track operations, increasing to 98.7% and 99.0%, respectively, at a depth of 6 m below the subgrade surface. This pattern demonstrates the effectiveness of the concrete box subgrade in attenuating acceleration, with attenuation rates surpassing 95%.

A comparative analysis of single- and double-track operations reveals that bidirectional operation induces a cumulative effect on vertical acceleration, resulting in somewhat greater acceleration than that during single-line operation. Interestingly, at equivalent depths, the attenuation rate during the bidirectional operation is marginally higher than that during the single-line operation. This comparison highlights the differential impact of operational modes on the dynamic behaviour of railway subgrades, particularly in terms of vertical acceleration and attenuation with depth.

#### 4.3.3 Ground acceleration with respect to the distance distribution

**Fig 19:**
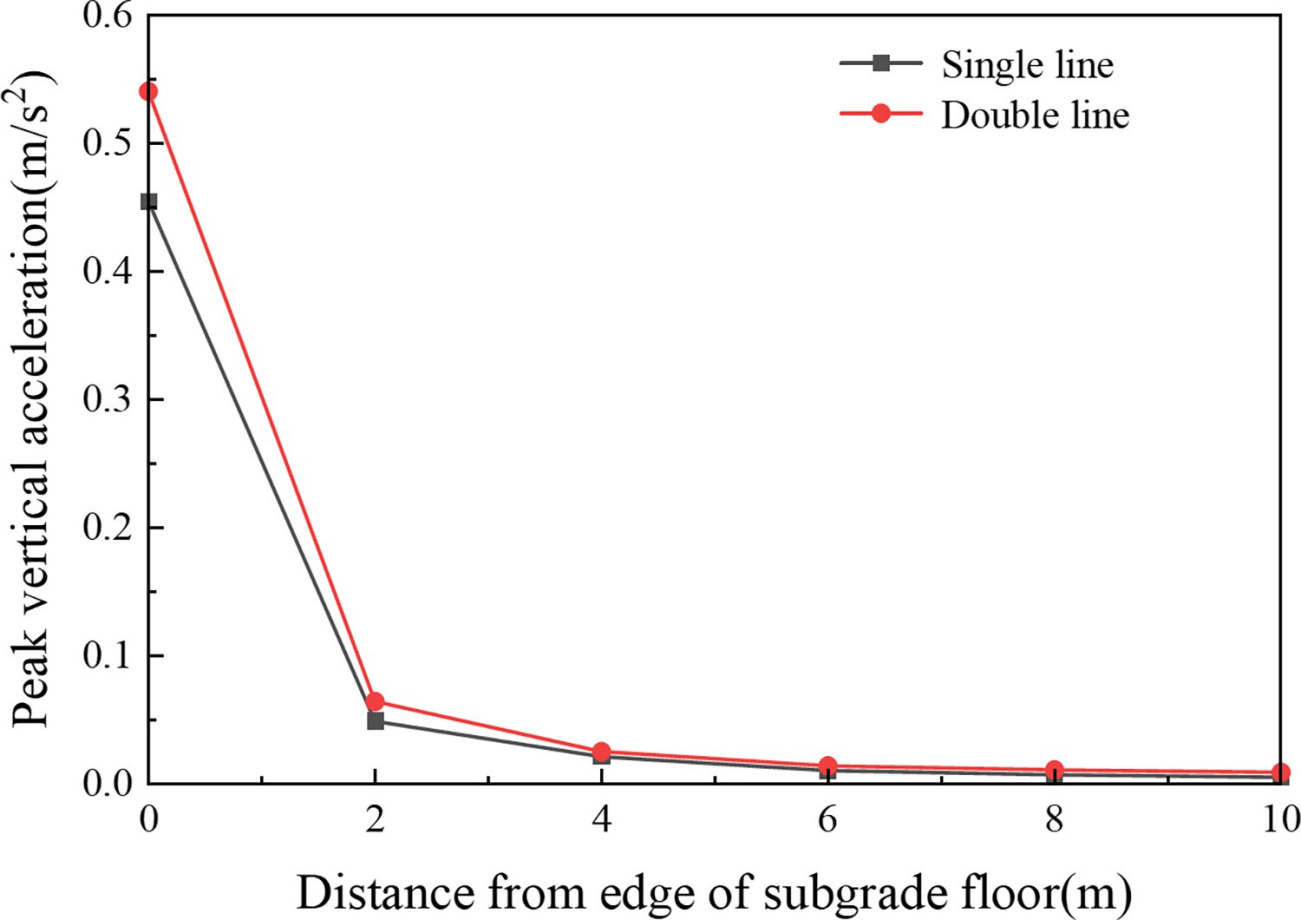


To investigate the impact of high-speed trains on the surrounding area, the variation in peak ground vertical acceleration with distance from the edge of the subgrade floor (L) was plotted, as shown in Fig 19. For both single-line and double-line operations, the peak ground vertical acceleration exhibited a general attenuation trend, with slightly greater acceleration observed during double-line operations. At the edge of the subgrade floor (L = 0 m), the peak ground accelerations were 0.455 m/s^2^ and 0.540 m/s^2^, respectively. At L = 4 m, the peak ground vibration accelerations decreased to 0.021 m/s^2^ and 0.025 m/s^2^, whereas at L = 10 m, the values further decreased to 0.005 m/s^2^ and 0.009 m/s^2^; this indicates that the dissipation of vibration energy due to the material and geometric damping properties of the soil results in a gradual reduction in ground vibration acceleration with increasing distance from the edge of the subgrade floor.

For both operational modes, the primary attenuation range of peak ground acceleration was between 0 m and 4 m, with an attenuation rate exceeding 95%. Beyond 4 m, the rate of decrease in peak acceleration was relatively slow; this suggests that the environmental vibrations induced by high-speed trains predominantly affect the area within 0 to 4 m from the edge of the subgrade floor.

## 5 Discussion

The concrete box subgrade, as an innovative embankment structure, has yet to be widely applied in practical engineering. Research on this type of subgrade has focused primarily on its structural form, stress characteristics [33, 34, 38], and settlement limits [35–37], whereas its dynamic response under train loads has received comparatively less attention. Furthermore, although extensive studies exist on the dynamic response of subgrades, most have concentrated on single-line models [12–14, 18]. However, most international high-speed railway systems adopt double-line embankment structures. The most critical loading condition in these systems occurs when trains travel in opposite directions on adjacent tracks, significantly increasing the likelihood of structural issues within the affected embankment sections. Therefore, scientific research on the dynamic response of high-speed railway concrete box subgrades is crucial to clarify their behaviour under such opposing traffic conditions.

The research findings indicate a lateral unevenness in the dynamic stress distribution on the surface of the concrete box subgrade, particularly within the concrete support layer. Under single-line operation, the vertical dynamic stress is primarily concentrated within the range of the operating line. Under opposing traffic conditions, the dynamic stress on the subgrade surface exhibits a "saddle-shaped" distribution, with the vertical acceleration attenuating mainly within the vertical plate. These results provide significant guidance for the further structural design and analysis of box subgrades. The load effect of double-line trains is not a simple superposition of loads; rather, stress redistribution occurs in the intersecting areas. Owing to the superposition effect, the dynamic response during double-line opposing operations is greater than that during single-line operations, with the dynamic stress increasing by 11.8% under a 300 km/h train load. Double-line opposing operations exacerbate the dynamic effects within the box subgrade, leading to greater damage to the subgrade. Therefore, special attention must be given during the design process.

As research on concrete box subgrades is still in its early stages, only the dynamic response under relatively simple conditions has been considered. Conditions such as uneven settlement and long-term loads have not yet been addressed, and these will be important areas for future research.

## 6 Conclusions

In the context of ballastless track systems for high-speed railways, the dynamic performance of the concrete box subgrade structure was analysed via a hybrid simulation approach that combines coupled dynamics with finite element modelling. This work presents an investigation of the distribution and attenuation of dynamic stress, displacement, and acceleration across the subgrade surface and investigated the effects of both single- and double-line bidirectional operation on the subgrade’s dynamic behaviour.

Under conditions of double-line opposing traffic, the dynamic stress displayed a lateral nonuniformity across the subgrade surface, particularly within the concrete base area, with peaks in the dynamic stress observed at the junctions where the vertical web of the concrete box subgrade meets the roof, indicating points of stress concentration. Beneath the rail position, an inverse increase in the vertical dynamic stress was noted within the depth range of the roof, which then rapidly diminished in the vertical web and roof, resulting in a notable reduction of 92.7% at the roof bottom. This demonstrates the significant diffusive effect of the concrete box subgrade on train loads and vibrations. The dynamic displacement on the subgrade surface, which displays a ’U-shaped’ lateral distribution from the centreline, reached a maximum of 0.320 mm. Through the depth of the concrete box subgrade, this displacement experienced a mere 3.3% attenuation, suggesting minimal vertical deformation overall, with the surface displacement primarily resulting from the underlying weak subsoil. The peak vertical acceleration recorded at the surface was 0.533 m/s^2^, which was predominantly attenuated within the vertical web depth and reached a reduction rate of 96.8%, whereas minimal attenuation was observed at the roof and floor depths. The primary attenuation range of peak ground acceleration lies between 0 and 4 m from the edge of the subgrade base. This area is the main area affected by environmental vibrations induced by high-speed trains.

In contrast, under single-line operation, the patterns of dynamic stress and displacement on the subgrade surface differed from those under double-line conditions, with peaks in stress and displacement occurring mainly on the side of the operational track. Comparing the two traffic conditions, the dynamic response of the concrete box subgrade under double-line opposing operation shows a marked increase, with the dynamic stress and acceleration amplitude rising by 5.7 kPa and 0.049 m/s^2^, respectively, equating to increases of 11.8% and 9.2%. Notably, the dynamic displacement at the subgrade surface under these conditions increased by 0.142 mm, or 80%, indicating that double-line opposing operations intensify the dynamic effects within the concrete box subgrade, thereby inflicting greater structural damage. Thus, particular attention must be given to controlling the dynamic displacement limits during design.

## Supporting information

S1 Data (XLSX)
